# Deciphering chemotherapy resistance: a novel apoptosis protein profile analysis in stage II colorectal cancer

**DOI:** 10.1136/bmjonc-2024-000460

**Published:** 2024-08-06

**Authors:** Zixiang Li, Leilei Fu

**Affiliations:** 1 Southwest Jiaotong University, Chengdu, Sichuan, China

**Keywords:** Colorectal cancer

 Colorectal cancer (CRC) is one of the leading causes of cancer-related deaths worldwide.^1^ Early-stage (I and II) surgical intervention is the standard treatment, with adjuvant chemotherapy used to minimize the risk of recurrence in high-risk stage II cases.^2^ The effectiveness of chemotherapy depends on various molecular and pathological factors, underscoring the necessity for customized therapeutic approaches. Both intrinsic and extrinsic apoptosis pathways, crucial in cell death and survival, play significant roles in cancer progression and treatment resistance. Previous attempts to link single apoptosis markers with chemotherapy outcomes have yielded inconsistent results, suggesting a complex interplay of factors.^3 4^

The study by Ginty F *et al* uses multiplexed imaging to analyse multiple apoptosis-related proteins at the cellular level in CRC.^5^ Their findings indicate that patients with a higher proportion of ‘persister’ cell profiles—characterised by low procaspase-3 and high X-linked inhibitor of apoptosis protein (XIAP)—face increased recurrence risks and gain limited benefits from adjuvant chemotherapy. This challenges traditional views by highlighting the heterogeneity in apoptosis signalling at the cellular level, potentially explaining why chemotherapy fails in certain cases.

This finding could help in identifying patients who are at higher risk of recurrence and might benefit from tailored therapeutic strategies that go beyond standard chemotherapy protocols. Given the link between specific protein clusters, especially those involving XIAP and SMAC, and increased recurrence risk,^6 7^ developing targeted inhibitors or modulators might be promising. For instance, therapies that inhibit XIAP expression or interfere with its interactions within the apoptosis pathway may enhance the susceptibility of cancer cells to apoptosis, thereby potentially improving the effectiveness of chemotherapy. Integrating proteomic data with genomic, transcriptomic and metabolomic data could yield a more comprehensive understanding of the molecular alterations associated with CRC progression and therapy resistance. This integrative approach might identify new therapeutic targets or biomarkers for predicting disease progression and treatment responses.

The study employs multiplexed immunofluorescence imaging to analyse apoptosis-related proteins at the single-cell level. This approach allows for a nuanced understanding of heterogeneity in protein expression within tumours.^8^ However, as Ginty F *et al* noted, there are limitations, including the possibility that the samples used may not fully represent the entire tumour’s heterogeneity, and the complex multimarker analysis required is difficult to implement in current clinical settings due to technological challenges. Additionally, reducing the number of markers simplifies the analysis but does not significantly enhance recurrence risk prediction, and issues with statistical significance after multiple testing further limit the results’ applicability. Thus, although the research offers new insights, further technological advances and validation are necessary before these findings can be applied clinically.

For patients with ‘persister’ cells that are resistant to chemotherapy, apoptosis sensitisers like venetoclax, which targets B-cell lymphoma 2 family proteins, may provide a new treatment strategy.^9^ Despite challenges such as drug-dosage adjustments and managing resistance, venetoclax has demonstrated significant potential in treating multidrug-resistant and recurrent cancers. When combined with other therapies, such as chimeric antigen receptor T-cell therapy or novel metabolic inhibitors, its therapeutic effects can be substantially enhanced.^10 11^

These insights into the apoptosis pathway are crucial for managing CRC, particularly in improving outcomes for stage II patients, where recurrence significantly impacts survival. Identifying biochemical markers for recurrence risk can improve prognostication and guide more personalised treatment decisions. The identification of ‘persister’ cell profiles that do not respond to chemotherapy could lead to more personalised treatment strategies, potentially sparing some patients from unnecessary and ineffective treatments.

In conclusion, specific cellular profiles can predict the efficacy of adjuvant chemotherapy in patients with stage II CRC, potentially leading to more targeted and effective treatment modalities that could ultimately improve patient outcomes.

## Data Availability

No data are available.
